# A Dual Systems Genetics Approach Identifies Common Genes, Networks, and Pathways for Type 1 and 2 Diabetes in Human Islets

**DOI:** 10.3389/fgene.2021.630109

**Published:** 2021-03-10

**Authors:** Simranjeet Kaur, Aashiq H. Mirza, Anne J. Overgaard, Flemming Pociot, Joachim Størling

**Affiliations:** ^1^Department of Translational T1D Research, Steno Diabetes Center Copenhagen, Gentofte, Denmark; ^2^Department of Pharmacology, Weill Cornell Medicine, New York, NY, United States; ^3^Pediatric Department E, University Hospital, Herlev, Denmark; ^4^Faculty of Health and Medical Sciences, University of Copenhagen, Copenhagen, Denmark; ^5^Department of Biomedical Sciences, University of Copenhagen, Copenhagen, Denmark

**Keywords:** type 1 diabetes, type 2 diabetes, genetics, network analysis, human islets

## Abstract

Type 1 and 2 diabetes (T1/2D) are complex metabolic diseases caused by absolute or relative loss of functional β-cell mass, respectively. Both diseases are influenced by multiple genetic loci that alter disease risk. For many of the disease-associated loci, the causal candidate genes remain to be identified. Remarkably, despite the partially shared phenotype of the two diabetes forms, the associated loci for T1D and T2D are almost completely separated. We hypothesized that some of the genes located in risk loci for T1D and T2D interact in common pancreatic islet networks to mutually regulate important islet functions which are disturbed by disease-associated variants leading to β-cell dysfunction. To address this, we took a dual systems genetics approach. All genes located in 57 T1D and 243 T2D established genome-wide association studies (GWAS) loci were extracted and filtered for genes expressed in human islets using RNA sequencing data, and then integrated with; (1) human islet expression quantitative trait locus (eQTL) signals in linkage disequilibrium (LD) with T1D- and T2D-associated variants; or (2) with genes transcriptionally regulated in human islets by pro-inflammatory cytokines or palmitate as *in vitro* models of T1D and T2D, respectively. Our *in silico* systems genetics approaches created two interaction networks consisting of densely-connected T1D and T2D loci genes. The “T1D-T2D islet eQTL interaction network” identified 9 genes (*GSDMB, CARD9, DNLZ, ERAP1, PPIP5K2, TMEM69, SDCCAG3, PLEKHA1*, and *HEMK1*) in common T1D and T2D loci that harbor islet eQTLs in LD with disease-associated variants. The “cytokine and palmitate islet interaction network” identified 4 genes (*ASCC2, HIBADH, RASGRP1*, and *SRGAP2*) in common T1D and T2D loci whose expression is mutually regulated by cytokines and palmitate. Functional annotation analyses of the islet networks revealed a number of significantly enriched pathways and molecular functions including cell cycle regulation, inositol phosphate metabolism, lipid metabolism, and cell death and survival. In summary, our study has identified a number of new plausible common candidate genes and pathways for T1D and T2D.

## Introduction

Type 1 (T1D) and 2 diabetes (T2D) are complex metabolic traits characterized by complete or relative insulin deficiency, respectively, due to destruction or failure of the β-cells in the pancreatic islets of Langerhans. In T1D, the β-cells are destroyed by both innate and adaptive immune mechanisms in which pro-inflammatory cytokines are believed to play key roles (Berchtold et al., 2016). During the process of immune-mediated β-cell killing, the β-cells are not just passive bystanders but actively participate in their own demise through the interface with the immune system via e.g., MHC class I expression and production of chemokines favoring islet infiltration of immune cells, and through their inherent “fragility” to immune damage (Soleimanpour and Stoffers, 2013; Mallone and Eizirik, 2020). β-cell failure in T2D may be caused by prolonged metabolic stress exerted by e.g., free fatty acids (FFA) such as palmitate, and by the persistent increased demand for insulin production due to peripheral insulin resistance ultimately leading to β-cell failure (Prentki and Nolan, 2006; Oh et al., 2018; Wysham and Shubrook, 2020). Hence, although different mechanisms lead to β-cell failure in T1D and T2D, the loss of functional β-cell mass is a common key mechanism and, in both cases the β-cells seem to play an active role (Eizirik et al., 2020).

Both T1D and T2D are polygenetic and disease risk is influenced by multiple genetic variants. To date, genome-wide association studies (GWAS) genotyping thousands of single nucleotide polymorphisms (SNPs) have established more than 50 and 200 risk loci for T1D and T2D, respectively (Barrett et al., 2009; Bradfield et al., 2011; Morris et al., 2012; Onengut-Gumuscu et al., 2015; Mahajan et al., 2018)^1^ Remarkably, the GWAS signals in T1D and T2D are starkly separated with only a few shared loci (Basile et al., 2014; Aylward et al., 2018) indicating vastly different genetic architectures. Among the few known common risk genes that have also been functionally validated is *GLIS3* which plays an important role in the β-cells by regulating proliferation and apoptosis (Nogueira et al., 2013; Wen and Yang, 2017). Based on its functional role, *GLIS3* has been suggested as an important predisposing factor of β-cell fragility in both forms of diabetes (Nogueira et al., 2013; Liston et al., 2017). Of note, the causal genetic variant(s) and gene(s) for most of the GWAS loci in T1D and T2D have not been identified. Better insight into the differences and putative commonalities of diabetes genetics may shed new light onto the pathogeneses of both diabetes forms.

Traditionally the gene located in closest physical proximity to the GWAS SNP in the disease locus has been considered the candidate risk gene (Slatkin, 2008). However, for complex polygenetic traits it has been reported that disease-associated SNPs are enriched for variants that have gene expression regulatory effects as determined by expression quantitative trait locus (eQTL) analyses (Westra and Franke, 2014; Fagny et al., 2017). eQTL analyses therefore represent an attractive way to link disease-associated SNPs to potential causal risk genes. Importantly, genetic variants can exert eQTL effects on genes that are physically distant to the disease-associated SNP underlining the complexity of disease genetics (Kumar et al., 2014). Based on this, it is plausible that many causal variants in T1D and T2D increase disease risk through changes in gene expression of nearby and distant genes. Notably, eQTLs can be highly tissue-specific emphasizing the necessity to examine eQTLs in relevant disease-affected tissue such as pancreatic islets in the case of T1D and T2D (Fagny et al., 2017).

In the present study, we aimed to take current knowledge of T1D and T2D genetics a step further by applying a systems genetics approach integrating GWAS data with human islet eQTLs and *in vitro* pathogenesis models to identify plausible causal risk genes, networks, and pathways shared between T1D and T2D at the pancreatic islet level. We identified a number of hitherto unreported common genes and pathways thereby advancing our understanding of shared genetic and pathogenic mechanisms in T1D and T2D. From our findings, novel hypotheses can be generated and tested in experimental disease models.

## Materials and Methods

### T1D and T2D Loci and Associated Genes

T1D loci; GWAS signals and candidate genes were retrieved from ImmunoBase^1^. ImmunoBase provides curated and integrated datasets of summary case/control association studies from 12 immunologically related human diseases including T1D originally targeted by the ImmunoChip consortium. T2D loci; GWAS signals and candidate genes were retrieved from Mahajan et al. (2018). All genes located ± 500 kb from GWAS-significant SNPs were extracted using bedtools (Quinlan and Hall, 2010). This window to retrieve loci-associated genes was selected based on published studies (Alasoo et al., 2019; Stacey et al., 2019). Previously pin-pointed/suggested causative candidate genes for each locus were retrieved as reported (Onengut-Gumuscu et al., 2015; Mahajan et al., 2018)^1^.

### Islet eQTLs and LD Analysis

Recently, Viñuela et al. (2020) profiled and genotyped human islet samples from 420 human organ donors as a part of Integrated Network for Systematic analysis of Pancreatic Islet RNA Expression (InsPIRE) consortium (Viñuela et al., 2020). The study aggregated previous islet studies and retrieved data from 196 individuals (Fadista et al., 2014; van de Bunt et al., 2015; Varshney et al., 2017). The samples were jointly mapped and reprocessed (median sequence-depth per sample ~60 M reads). We retrieved both exon- and gene-based islet eQTLs from this study to identify islet eQTLs associated with T1D-T2D loci.

Islet eQTLs in linkage disequilibrium (LD) (*r*^2^ ≥ 0.8) with nominally associated disease variants (both T1D and T2D variants) were identified using SNIPA (Arnold et al., 2015). The variant set used for LD calculation was 1,000 Genome, Phase 3 v5 (GRCh37 genome build), European population. For T1D and T2D, 20,669 and 5,270 nominally associated SNPs were obtained (*p* < 0.05), respectively. The summary statistics from a BMI-adjusted European dataset were used to retrieve T2D SNPs from Mahajan et al. (2018).

All the islet eQTL variants were annotated with Islet Regulome chromatin classes (including islet enhancers, promoters, and CTCF binding sites) retrieved from Mularoni et al. (2017) and Miguel-Escalada et al. (2019) using intersectBed feature of Bedtools (Quinlan and Hall, 2010). The T1D/T2D variants in LD with islet eQTLs were also annotated with islet regulome features.

Total RNAseq datasets from FACS-purified human α-, β-, and exocrine cells from 8 organ donors without diabetes were retrieved from GEO (GSE50386 and GSE76268) (Bramswig et al., 2013; Ackermann et al., 2016). The datasets included libraries that were single-end sequenced to 100 bp on an Illumina hiSeq2000. The raw fastq files were trimmed, cropped, and adapters removed using Trimmomatic v.0.36 (Bolger et al., 2014). The filtered reads after pre-processing (trimming and adapter removal) were aligned to a human genome (GRCh38) using tophat 2.1 (Trapnell et al., 2009) using the following parameters: Library-type = fr-firststrand, no-coverage-search, m 2, p 10. The raw read counts at gene level were calculated using htseqcount and further normalized to counts per million (CPM) and logCPM in EdgeR (Robinson et al., 2010).

### Genes Transcriptionally Modified by Cytokines or Palmitate in Human Islets

The differentially expressed genes after cytokine (IL-1β + IFNγ) or palmitate exposure for 48 h in human islets were retrieved from GEO datasets GSE35296 and GSE53949, respectively (Eizirik et al., 2012; Cnop et al., 2014). Both datasets included five human islet preparations obtained from organ donors without diabetes, treated, and handled under similar conditions, with comparable human islet collection and handling protocols. In both studies, paired-end total RNA-sequencing was performed using polyA-selected mRNA and the datasets were processed using similar methods. Briefly, the authors mapped the paired end reads to human genome (GRCh37) using Genomic Multitool (GEM) suite (https://bio.tools/gemmapper) and transcripts were quantified into RPKM values using Flux Capacitor (http://flux.sammeth.net) (Eizirik et al., 2012; Cnop et al., 2014). The differentially expressed genes were identified using Fisher's exact test and *p*-values were corrected using Benjamini-Hochberg method. A difference in gene expression was considered significant if the adjusted *p* < 0.05 and if the expression changed significantly in one direction in at least four out of the five islet preparations (Eizirik et al., 2012; Cnop et al., 2014). In total 3,019 genes were found to be modulated by cytokines whereas 1,236 genes were modified by palmitate. Of these, 494 genes were regulated by both cytokines and palmitate.

### PPI Network and Pathway Analysis

ToppCluster within ToppGene Suite (Chen et al., 2009) was used to identify protein-protein interactions (PPIs) between the T1D and T2D loci genes. Cytoscape v3.7.0 (http://www.cytoscape.org) (Smoot et al., 2011) was used to visualize the PPI network. A network topological analysis was performed using NetworkAnalyzer v2.7 which is a part of Cytoscape to assess various topological features. For every node in a network, NetworkAnalyzer computes its degree, the number of self-loops, and a variety of other parameters.

Ingenuity pathway analysis (IPA, Qiagen Inc.) was used to predict the downstream effects of the selected genes from the PPI networks. IPA has the most comprehensive, manually curated QIAGEN Knowledge Base that includes data derived from “omics” experiments including RNAseq, small RNAseq, metabolomics, proteomics, microarrays, and small-scale experiments from published studies^2^. IPA core analysis was performed to identify enriched pathways and molecular and cellular functions for the T1D and T2D loci genes in the PPI networks.

Pathway analysis was also performed using ClueGO plug-in v2.5.3 (Bindea et al., 2009) in Cytoscape. ClueGO integrates GO terms and pathways into a PPI network and creates a functional annotation map that represents the associations between terms. Pathway based clustering was performed with following settings: minimum number of genes within each cluster = 3, pathway network connectivity measure (κ score) = 0.4. The κ score defines the term-term interrelations and creates functional groups based on shared genes between the terms. The *p*-values were calculated using two-sided hypergeometric test and adjusted using Bonferroni step-down method. The minimum percentage of genes and terms for group merge was 50%. KEGG, Reactome, and WikiPathway annotations were used for pathway-based enrichment analyses in ClueGO.

STRING database and STRING enrichment app (Doncheva et al., 2019) in Cytoscape were used for expanding the network for the selected shared genes. The extended network in STRING was created using the following parameters: A confidence score cutoff of 0.5, selectivity of interactors 0.5 and the total number of interactors to expand the network was set to 50. The KEGG and Reactome pathway annotations were used to perform STRING enrichment analysis. The significant pathways were selected based on an FDR value <0.05.

## Results

### Selection and Integration of T1D and T2D Loci Genes and Islet eQTLs

**Figure 1:**
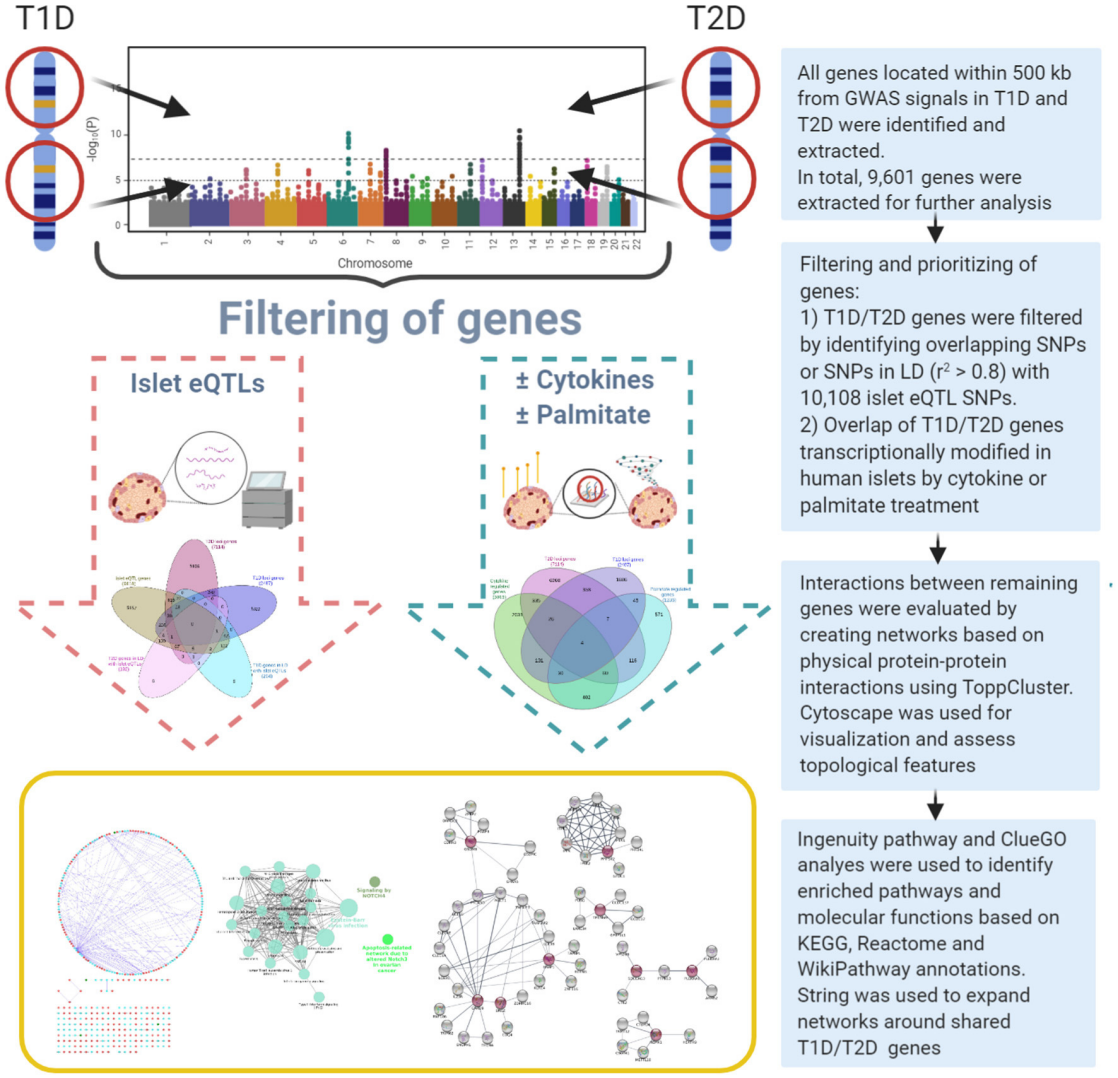
Overview of the dual systems genetics approach applied.

**Figure 2:**
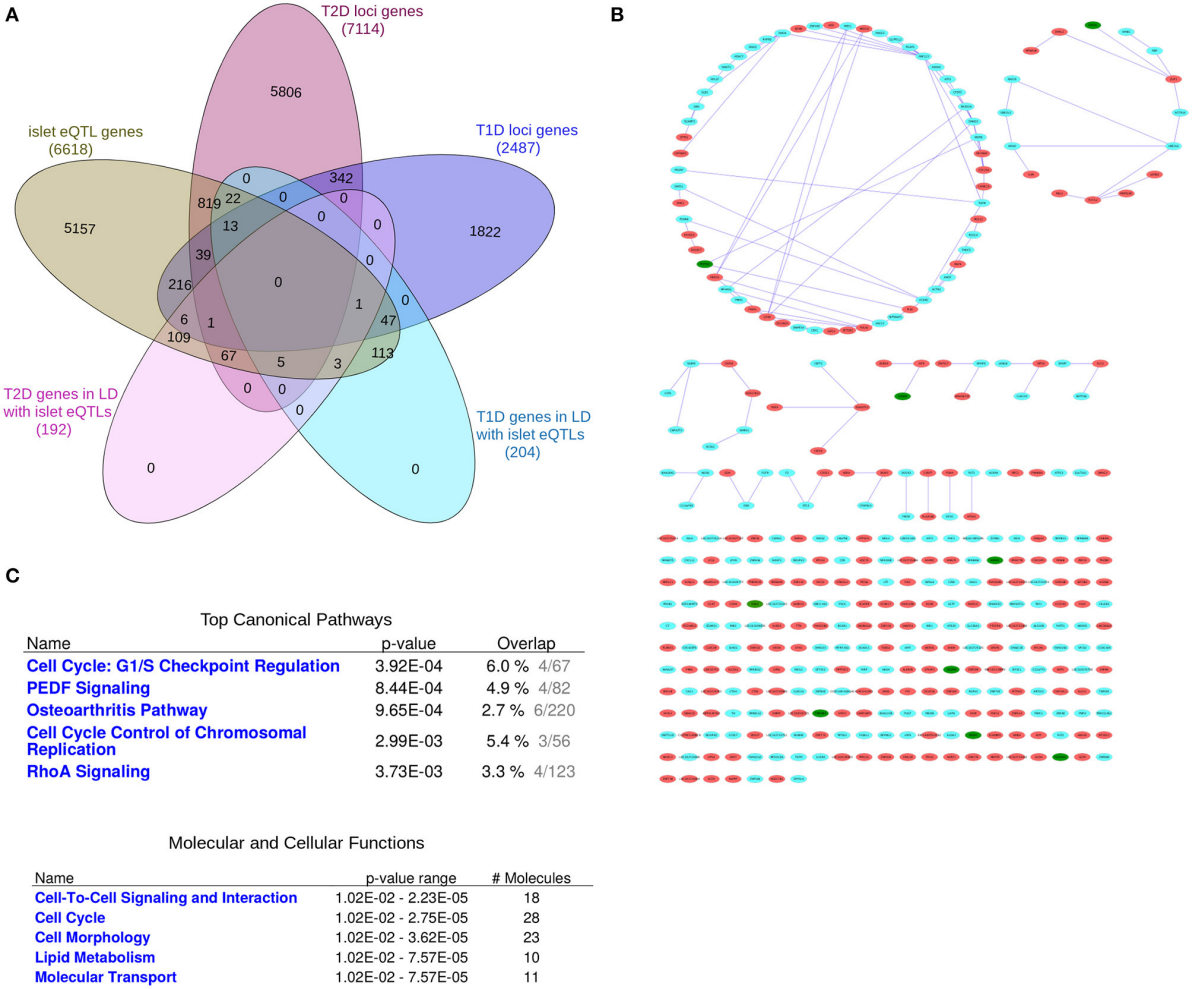
The “T1D-T2D islet eQTL interaction network.” The “T1D-T2D islet eQTL interaction network” is derived from islet eQTLs in linkage disequilibrium (LD ≥0.8) with nominally associated disease variants (both T1D and T2D variants). **(A)** Venn diagram of the genes filtered for. **(B)** The 'T1D-T2D islet eQTL interaction network' as visualized by Cytoscape. T1D (*n* = 204) and T2D genes (*n* = 192) are shown as cyan and red nodes, respectively. The edges (blue lines) represent the physicial interactions between the nodes. The network consists of 361 nodes, of which 9 are shared (shown in green). **(C)** Functional annotation of the “T1D-T2D islet eQTL interaction network” (117 nodes with a node degree ≥1) based on IPA analysis. Top canonical pathways and molecular and cellular processes are shown for the selected nodes.

A systems genetics approach was applied to pinpoint likely causal T1D and T2D risk genes and to examine their putative interactions in joint networks in human islets—the common “diseased tissue” in T1D and T2D (Figure 1). We divided our overall approach into two sub-approaches integrating; (1) T1D and T2D loci genes with human islet eQTL data, and (2) T1D and T2D loci genes with cytokine- or palmitate-modified human islet gene expressional changes. First, all genes located within ± 500 kb from 107 and 380 genome-wide significant signals for T1D and T2D, respectively, were extracted from publicly available data from ImmunoBase and Mahajan et al. (2018)^1^. These signals corresponded to 57 T1D and 243 T2D genomic loci of which 5 were overlapping. The genomic loci were defined based on conditionally independent signals that reach the GWAS significance ± 500 kb surrounding the lead SNP (Mahajan et al., 2018)^1^. If the minimum distance between any distinct signals from two separate loci was <500 kb, additional conditional analysis taking both regions (encompassing ± 500 kb from both ends) were performed to assess the independence of each signal (Mahajan et al., 2018). Of the total 403 identified distinct signals by Mahajan et al. 380 remained after excluding 23 signals that were not amenable to fine mapping (Mahajan et al., 2018). In total, 2,487 and 7,114 genes were retrieved for the T1D and T2D loci, respectively (Figure 2A).

Leveraging on a study by Vinuela and colleagues (Viñuela et al., 2020) that profiled gene expression and performed genotyping of human islets from 420 individual donors, we retrieved islet eQTLs. Both exon and gene-level *cis*-eQTLs corresponding to 4,312 and 6,039 genes, respectively (FDR < 1%; *cis* defined as within 1 Mb of the transcription start site [TSS]), were combined that resulted in a total of 10,108 islet eQTL associations for 6,618 genes (Table 1.1 in Supplementary File 1). The majority of the islet eQTL signals were associated with protein-coding genes (*n* = 9,627), while a much lower fraction was associated with long non-coding RNA genes (*n* = 842). We annotated the islet eQTL variants with Islet regulome features to identify enrichment for islet regulatory elements including islet enhancers, promoters, open chromatin regions, and CTCF binding sites etc. Only 12% (1,282 SNPs) of the islet eQTLs showed overlap with islet regulatory features, whereas the majority of the islet eQTL SNPs did not show any overlap (Table 2.1 in Supplementary File 2).

To further filter and prioritize the islet eQTL genes, we performed LD analysis to identify T1D and T2D GWAS SNPs that either themselves have islet eQTL effects or are in strong LD (*r*^2^ > 0.8) with islet eQTL SNPs. For this analysis, we included all nominally associated SNPs for both T1D and T2D with a *p* < 0.05. Using a LD cutoff of *r*^2^ > 0.8, 242,191 proxy SNPs were retrieved for the 10,108 islet eQTL SNPs.

For the T1D loci, 247 islet eQTLs SNPs (associated with 204 genes) were in LD with 1,735 T1D-associated SNPs. Of these, 55 of the T1D-associated SNPs directly acted as islet eQTL signals for T1D loci genes (Table 1.2 in Supplementary File 1). For the T2D loci, 223 islet eQTLs SNPs (associated with 192 genes) were in LD with 176 T2D SNPs. Of these, 19 of the T2D-associated SNPs directly acted as islet eQTL signals for T2D loci genes (Table 1.3 in Supplementary File 1). The annotation of these T1D and T2D SNPs in LD with islet eQTL SNPs with islet regulome features and Variant Effect Predictor (VEP) are shown in (Tables 2.2, 2.3 in Supplementary File 2).

### Generation of a Common T1D-T2D Islet eQTL Interaction Network Based on Genes in LD With Disease Variants

We created a “T1D-T2D islet eQTL interaction network” based on the genes with islet eQTLs in LD with nominally associated T1D and T2D SNPs, i.e., the 204 T1D and 192 T2D loci-associated genes (Figures 2A,B).

Figure 2B (see Supplementary File 3 for a high resolution image) shows the generated network which consists in total of 361 nodes, of which 9 are shared between T1D and T2D (shown as green nodes). These shared genes are *GSDMB, CARD9, DNLZ, ERAP1, PPIP5K2, TMEM69, SDCCAG3, PLEKHA1*, and *HEMK1* and are covered in detail in the following section.

Pathway-based functional annotation was performed on the 117 nodes with a node degree ≥1 (i.e., with at least one physical interaction partner) in the “T1D-T2D islet eQTL interaction network.” The top canonical pathways based on IPA pathway analysis included “Cell Cycle: G1/S checkpoint regulation,” “PEDF Signaling,” “Osteoarthritis Pathway,” “Cell cycle control of chromosomal regulation,” and “RhoA signaling” (Figure 2C). The identified molecular and cellular processes included “Cell-to-cell signaling and interaction,” “Cell cycle,” “Cell morphology,” and “Lipid metabolism.”

We also performed ClueGO pathway analysis of the “T1D-T2D islet eQTL interaction network” which identified 10 highly significant pathways that grouped into 5 clusters (Figure 1 in Supplementary File 1). The representative pathways and genes for these 5 clusters were “Sphingolipid metabolism” (with 3 genes involved: *CERS2, GBA, GLB1*), “Transcriptional regulation of white adipocyte differentiation” (with 4 genes involved: *CDK8, MED1, MED28, MED31*), “G1 to S cell cycle control” (with 3 genes involved: *CDC25A, POLA2, PRIM1*), “Ethanol effects on histone modifications” (with 4 genes involved: *ACSS2, ATF2, MED1, HDAC7*) and “Chromosomal and microsatellite instability in colorectal cancer” (with 6 genes involved: *RHOA, SMAD3, TCF7L2, CDK8, ATF2, PLEC*). All the 10 significant pathways are listed in Table 1.4 in Supplementary File 1 along with their clusters and *p*-values.

### Extended Network of Shared Genes and Pathway Analysis

The 9 shared genes between T1D and T2D found in the “T1D-T2D islet eQTL interaction network” (Figure 2B) were explored further in relation to; (1) their shared eQTL signals for T1D and T2D; (2) their neighboring interacting partners; and (3) their associated pathways. Table 1 lists the 9 shared genes and their islet eQTL associations with T1D- and T2D-associated SNPs. The genes with islet eQTLs in LD with highly significant (GWAS *p* < 2E-08) T1D- and T2D-associated SNPs were *GSDMB* and *CRAD9* (Table 1).

**Table 1:** Islet eQTL SNPs in LD with disease-associated SNPs for the 9 shared genes within the “T1D-T2D islet eQTL interaction network.”

**slet eQTLs**							**T1D-associated SNP in LD (r2>0.8)**		**T2D-associated SNP in LD(r2>0.8)**	
**Gene name**	**eQTL SNP**	**A1**	**A2**	**MAF**	**chrSNP**	**StartSNP**	**SNP**	**P-value**	**SNP**	***P***
HEMK1	rs12493985	T	G	0.14	3	50544715	rs1034405	2.39E-03	rs1034405	0.022
GSDMB	**rs870829**	A	C	0.42	17	38068382	**rs870829**	2.42E-08		
	rs12939565	A	T	0.47	17	38038389	rs12453507	1.05E-08	rs11557467	0.019
ERAP1	**rs7063**	A	T	0.29	5	96110211	**rs7063**	0.014		
	rs146341958	C	T	0.13	5	96125159			rs72773968	0.007
PPIP5K2	rs1898673	G	C	0.33	5	102293380	rs3776855	0.016	rs34813	0.00064
	rs27489	C	T	0.28	5	102555746	rs3776855	0.016	rs34813	0.00064
TMEM69	rs28597977	A	G	0.32	1	46181206	rs6694302	0.040	rs28375469	0.049
DNLZ	rs57052773	T	C	0.04	9	139385701	rs78270318	0.012	rs3812561	0.002
	rs28679497	G	A	0.28	9	139246594			rs60980157	2.02E-15
	rs4442263	C	T	0.04	9	139322775	rs78270318	0.012	rs3812561	0.002
SDCCAG3	rs34619169	G	A	0.27	9	139327277	rs11146021	0.021	rs3812594	0.0051
CARD9	rs57052773	T	C	0.04	9	139385701	rs78270318	0.012	rs3812561	0.002
	rs61386106	G	A	0.28	9	139246768			rs60980157	2.02E-15
	rs4442263	C	T	0.04	9	139322775	rs78270318	0.012	rs3812561	0.002
PLEKHA1	rs4752689	G	A	0.4	10	124131176			rs1045216	9.80E-06
	rs71486610	G	C	0.49	10	124134803	rs2280141	0.030		
							rs7097701	0.0270		

*The table shows the 9 shared genes in the “T1D-T2D islet eQTL interaction network.” eQTLs that are disease-associated SNPs themselves are shown in bold. T1D/T2D SNPs in LD with islet eQTL SNPs are listed. In case of multiple disease-associated SNPs in LD with an eQTL SNP, the most significant disease-associated SNP is listed. The disease-associated SNPs with GWAS significance are highlighted in bold*.

**Figure 3:**
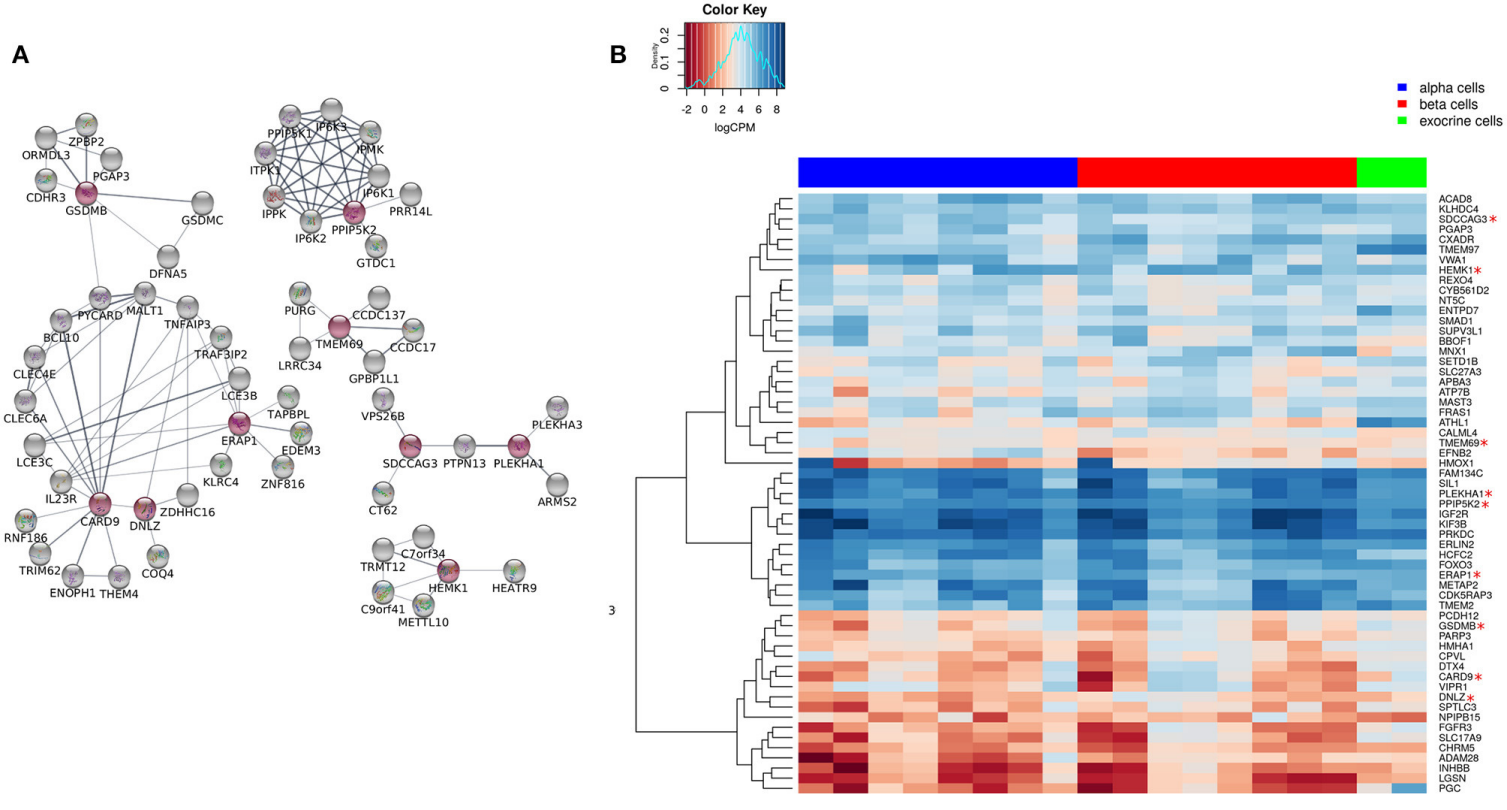
Extended network of the 9 shared genes within the “T1D-T2D islet eQTL interaction network.” **(A)** The 9 shared genes within the “T1D-T2D islet eQTL interaction network” are shown as red nodes. The network was extended based on physical interactions to allow 50 neighboring genes (shown in gray nodes) using STRING app in Cytoscape. **(B)** Expression of the genes within the extended network (*n* = 59) in FACS-purified α, β, and exocrine cells derived from human islets. The heatmap shows unsupervised clustering of log2CPM values for α-cells (*n* = 8), β-cells (*n* = 8), and exocrine cells (*n* = 2) samples. The clustering was done using Euclidean distance with complete linkage method.

We extended the network of the 9 shared T1D/T2D genes by including neighboring genes to create a larger network allowing identification of their associated pathways. Figure 3A shows the extended network of the 9 shared genes. The extended network was expanded by allowing a maximum of 50 interactors shown in gray nodes (Figure 3A). The STRING enrichment analysis identified 17 significant pathways and an overall PPI enrichment score of 1.0E-16. A PPI enrichment score <0.05 indicates that the proteins are more likely to be biologically connected as a group. The top 5 pathways for the extended network included “inositol phosphate metabolism,” “synthesis of pyrophosphates in the cytosol,” “phosphatidylinositol signaling system,” “synthesis of IPs in the nucleus,” and “c-type lectin receptors (CLRs)” (Table 2). We then analyzed the expression of the genes in the extended network using RNAseq data from FACS-purified α-, β-, and exocrine cells derived from human islets. Figure 3B shows a heatmap of the expression values of the genes in the three cell types.

**Table 2:** Pathway-based functional annotation of the extended network of the 9 shared genes within the “T1D-T2D islet eQTL interaction network.”

**Annotation Source**	**# Background genes**	**# Genes**	**Description**	**Genes**	**FDR Value**
Reactome	48	8	Inositol phosphate metabolism	ITPK1, IPPK, IP6K2, IPMK, IP6K3, PPIP5K1, IP6K1, **PPIP5K2**	4.31E-10
Reactome	10	6	Synthesis of pyrophosphates in the cytosol	ITPK1, IPPK, IP6K3, PPIP5K1, IP6K1, **PPIP5K2**	4.31E-10
KEGG	97	8	Phosphatidylinositol signaling system	ITPK1, IPPK, IP6K2, IPMK, IP6K3, PPIP5K1, IP6K1, **PPIP5K2**	2.81E-08
Reactome	4	4	Synthesis of IPs in the nucleus	IPPK, IP6K2, IPMK, IP6K1	1.65E-07
Reactome	134	6	C-type lectin receptors (CLRs)	PYCARD, CLEC4E, MALT1, BCL10, **CARD9**, CLEC6A	9.48E-05
Reactome	94	4	CLEC7A (Dectin-1) signaling	PYCARD, MALT1, BCL10, **CARD9**	0.0042
Reactome	6	2	CLEC7A/inflammasome pathway	PYCARD, MALT1	0.0042
Reactome	53	3	Synthesis of PIPs at the plasma membrane	PLEKHA3, **PLEKHA1**, PTPN13	0.0089
Reactome	54	3	Nucleotide-binding domain, leucine rich repeat containing receptor (NLR) signaling pathways	PYCARD, **CARD9**, TNFAIP3	0.0089
Reactome	2032	15	Metabolism	PLEKHA3, ITPK1, ENOPH1, IPPK, IP6K2, THEM4, **PLEKHA1**, IPMK, C9orf41, ORMDL3, PTPN13, IP6K3, PPIP5K1, IP6K1, **PPIP5K2**	0.009
Reactome	1925	14	Immune System	PYCARD, TRIM62, ERAP1, CLEC4E, MALT1, IL23R, IP6K2, THEM4, BCL10, **CARD9**, CLEC6A, ORMDL3, PTPN13, TNFAIP3	0.0145
Reactome	84	3	PI Metabolism	PLEKHA3, **PLEKHA1**, PTPN13	0.0202
KEGG	73	3	Inositol phosphate metabolism	ITPK1, IPPK, IPMK	0.0241
KEGG	93	3	NF-kappa B signaling pathway	MALT1, BCL10, TNFAIP3	0.0241
KEGG	172	4	Tuberculosis	CLEC4E, MALT1, BCL10, **CARD9**	0.0241
Reactome	26	2	Dectin-2 family	CLEC4E, CLEC6A	0.0258
Reactome	35	2	NOD1/2 Signaling Pathway	**CARD9**, TNFAIP3	0.0413

*The functional annotations of the extended network of the 9 shared genes are shown in the table. KEGG and Reactome pathway annotations were used for the pathway enrichment analysis using the STRING app in CytoScape. The 9 shared genes associated with the enriched pathways are highlighted in bold*.

### Generation of a Common T1D-T2D Islet Interaction Network Based on Cytokine- and Palmitate-Regulated Loci Genes

**Figure 4:**
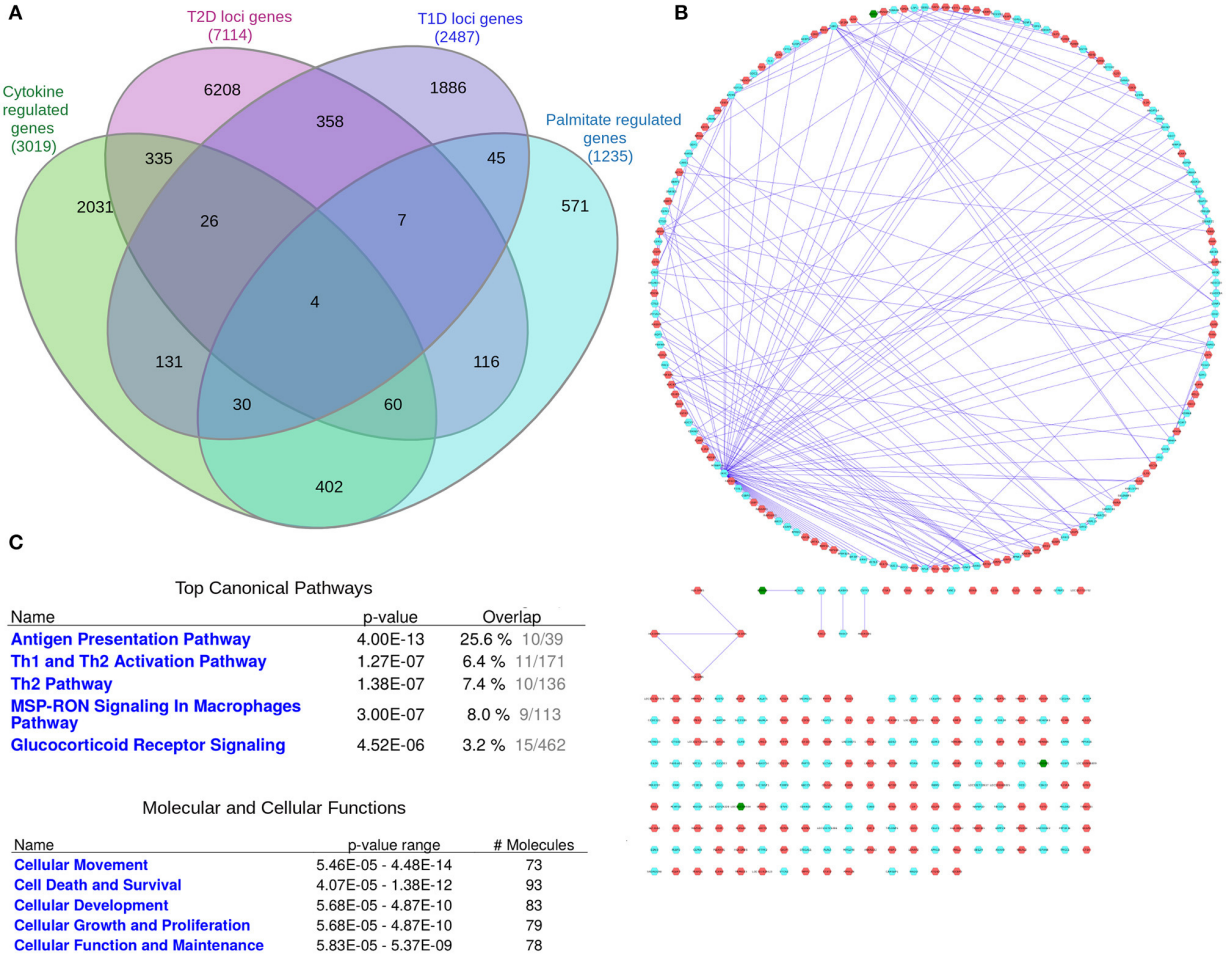
The “cytokine and palmitate islet interaction network.” The “cytokine and palmitate islet interaction network” is derived from overlap of T1D and T2D loci genes with cytokine- and/or palmitate-regulated genes. **(A)** Venn diagram of the genes filtered for. **(B)** The “cytokine and palmitate islet interaction network” as visualized by Cytoscape. The T1D loci genes subject to cytokine regulation (*n* = 191) and T2D loci genes subject to palmitate regulation (*n* = 187) are shown in cyan and red nodes, respectively. The edges (blue lines) represent the physicial interactions between the nodes. The network consists of 372 nodes, of which 4 are shared (shown in green). **(C)** Functional annotation of the “cytokine and palmitate islet interaction network” (181 nodes with node degree ≥1) based on IPA analysis. Top canonical pathways and molecular and cellular processes are shown for the selected nodes.

As eQTLs may not be present under basal, non-disease conditions, but only in the disease state or the phase preceding disease, we sought to take an additional approach to investigate interactions between islet expressed T1D and T2D loci genes. We therefore next created a network of T1D and T2D loci genes whose expression in human islets is modulated by pro-inflammatory cytokines as an *in vitro* model of a T1D environment and/or by the FFA palmitate as an *in vitro* model of a T2D environment using published RNAseq datasets (Eizirik et al., 2012; Cnop et al., 2014) (Figures 4A,B). In total, cytokines modulated the expression of 191 T1D loci genes whereas palmitate modulated the expression of 187 T2D loci genes. Interestingly, among these, 4 genes (*ASCC2, HIBADH, RASGRP1, and SRGAP2*) were commonly regulated by cytokines and palmitate and were also located in shared T1D and T2D loci (Table 1.5 in Supplementary File 1). Figure 4B (see Supplementary File 4 for a high resolution image) depict the derived network with a total of 372 nodes, the 4 shared genes are shown in green nodes.

IPA pathway analysis of the “cytokine and palmitate islet interaction network” identified “Antigen presentation” and “Th1 and Th2 activation” as top canonical pathways (Figure 4C). The top molecular and cellular processes included “Cellular movement,” “Cell death and survival,” and “Cell proliferation and growth.”

Functional annotation of the “cytokine and palmitate islet interaction network” using ClueGO revealed 3 clusters of 24 highly significant pathways (Figure 2 and Table 1.6 in Supplementary File 1). The representative terms and genes for these 3 clusters are “Signaling by NOTCH4” (with 6 genes involved: *ACTA2, FBXW7, NOTCH2, PSMB1, PSMB8, PSMB9*), “Apoptosis-related network due to altered Notch3 in ovarian cancer” (with 5 genes involved: *APOE, AXIN1, ERBB3, ERN1, IL7R*) and “Epstein-Barr virus infection” (with 35 genes involved: *HLA-DMA, HLA-DMB, HLA-DPA1, HLA-DRA, HLA-DRB5, ITGB3, TAP1, TAP2, TUBA4A, CIITA, IL2RA, IL7R, NOTCH2, RARA, PTPRN2, EEF1A2, SOCS1, ICAM1, KRT40, CEBPG, CTSD, LSP1, IKBKE, KPNA2, OAS3, ADCY5, CDKN2C, FOSL1, MYC, DDB2, TNFAIP3, RAC2, AP1B1, AP2M1, PSMB9*).

## Discussion

In this study, we employed a systems genetics approach integrating RNAseq data, eQTL signals and cytokine/palmitate-regulated genes to look for PPIs between probable causal risk genes in T1D and T2D GWAS loci at the human pancreatic islet level. We were able to create a PPI network that contained interactions between multiple T1D and T2D loci genes that associated with SNPs in LD with islet eQTL SNPs. IPA pathway analysis of the interacting nodes pointed toward important cellular processes such as regulation of cell cycle processes. Considering that loss of functional β-cell mass is a key mechanism in both T1D and T2D, it is plausible to think that disease-associated variants linked to altered gene expression of genes involved in cell cycle control could negatively affect the replicative capacity of the β-cells thereby favoring a loss of functional β-cell mass.

Interestingly, we identified 9 shared genes within the “T1D-T2D islet eQTL interaction network” and an extension of the network surrounding these shared genes revealed highly interconnected nodes that are putatively involved in regulating common processes leading to either type of disease. Among the 9 shared genes (*HEMK1, GSDMB, ERAP1, PPIP5K2, TMEM69, DNLZ, SDCCAG3, CARD9, and PLEKHA1*), two of them, *GSDMB* (gasdermin B) and PLEKHA1 (pleckstrin homology domain-containing family A member 1) were previously identified as candidate genes for T1D (Morris et al., 2012) and T2D (Mahajan et al., 2018), respectively. *GSDMB* and *CARD9* (caspase recruitment domain family member 9), both have implications in the inflammatory pathways leading to apoptosis (Hara et al., 2007; Ruan, 2019). Three genes encode for enzymes with different functions, *ERAP1* (endoplasmic reticulum aminopeptidase 1) an amino peptidase involved in the processing of HLA class I-binding precursors (Rock et al., 2002), a histidine acid phosphatase, *PPIP5K2* (diphosphoinositol pentakisphosphate kinase 2), regulating bioenergetic homeostasis (Nair et al., 2018), and *HEMK1* (methyl transferase family member 1), responsible for the methylation of glutamine residues. Further, *PLEKHA1* (pleckstrin homology domain containing A1) is involved in signaling complexes in the plasma membrane. *SDCCAG3* (serologically defined colon cancer antigen 3) is potentially related to protein trafficking and secretion (Neznanov et al., 2005).

The enriched pathways for the extended network of the 9 shared genes included interesting categories such as “inositol phosphate metabolism,” “immune system,” “inflammasome pathway,” and “NOD1/2 signaling.” Broadly speaking, most if not all these pathway functions seem rational in terms of regulating cellular mechanisms that could be important for diabetes at the islet level. For instance, with regard to “inositol phosphate metabolism,” it is well-recognized that inositol phosphate compounds are intimately involved in the stimulus-secretion coupling process in β-cells through the regulation of calcium signaling (Barker et al., 2002). Remarkably, there was as many as 6 enriched pathways in total related to inositol in the extended network inferring that inositol signaling and metabolism may play prime roles in both T1D and T2D.

Identifying protein complexes from PPIs is an important area of research for gaining insights into genetic pathways and identification and prioritization of disease genes (Lage et al., 2007; Taylor and Wrana, 2012). An increasing number of studies have employed PPI networks to explore the molecular basis of complex diseases (Oti et al., 2006; Bergholdt et al., 2007; Lage et al., 2007; Jaeger and Aloy, 2012). Genes causing the same or similar diseases tend to lie close to one another in a network of PPIs or functional interactions and display a high degree of connectivity (Oti et al., 2006; Vanunu et al., 2010). Previous studies combining PPIs and genetic interactions predicted disease genes for genetically heterogeneous diseases and proved helpful in identifying associations between disease genes and other genes for specific protein complexes (Oti et al., 2006; Bergholdt et al., 2007, 2009; Lage et al., 2007; Vanunu et al., 2010).

An important part of our study design of the first approach was the gene filtering based on regulatory islet eQTLs. Lappalainen et al. (2013) provided a detailed landscape of regulatory SNPs in 1,000 Genomes data and demonstrated how eQTL data can be used to identify potential causal variants. In a recent study, Fagny et al. (2017) constructed tissue-level eQTL networks in 13 human tissues and observed tissue-specific regulatory roles of common variants and their collective impact on biological pathways underlining the necessity to look for eQTLs in the specific tissue of interest, i.e., in islets in our current study, to obtain meaningful and (patho)physiologically relevant information.

In our second part of analyses, we also selected for loci genes that were subject to differential regulation by cytokines and/or palmitate as *in vitro* models of T1D and T2D. This analysis revealed 4 commonT1D and T2D loci genes (*ASCC2, HIBADH, RASGRP1, and SRGAP2*) that are all regulated by both cytokines and palmitate in human islets. *RASGRP1* (RAS guanyl releasing protein 1) was previously identified as a candidate gene for both T1D and T2D (Mahajan et al., 2018), and *ASCC2* (activating signal cointegrator 1 complex subunit 2) and *SRGAP2* (SLIT-ROBO Rho GTPase-activating protein 2) were identified as T2D candidate genes (Mahajan et al., 2018)^1^. Interestingly, two of the genes, *ASCC2* and *HIBADH*, were also associated with islet eQTLs, and even more remarkably, we found that *ASCC2* eQTL SNPs were in strong LD with T1D-associated SNPs (data not shown). *ASCC2* is involved in ubiquitin binding activity which might be responsible for commonly regulating β-cell function in human islets and contributing to both T1D and T2D (López-Avalos et al., 2006), which deserves further investigation in future studies. *HIBADH* (3-hydroxyisobutyrate dehydrogenase) has been previously implicated in insulin resistance and risk of incident type 2 diabetes and gestational diabetes mellitus (Nilsen et al., 2020). Although the selection of genes based on their regulation by cytokines and/or palmitate does not necessarily identify causal genes, but merely identifies genes whose expression level correlate with cytokine/palmitate exposure. It is also important to keep in mind that the genes observed to be differentially expressed at a specific time point only reflect a snapshot of the gene regulatory effects exerted by cytokines and palmitate. Despite these drawbacks, we do believe that this approach is a valid alternative approach to the eQTL/LD selection criteria in our first part of the analyses.

Our finding that risk genes for T1D and T2D interact in shared networks at the islet level supports the concept that despite the overall lack of genetic commonality in T1D and T2D, and that different mechanisms underlie the loss of functional β-cell mass in T1D and T2D, at least some candidate risk genes of both diabetes forms seem to cooperate in common pathways to regulate various islet processes that could be relevant for promoting disease. The common networks identified by our analyses adds to our current knowledge and may offer an opening to pinpoint potential commonality between T1D and T2D. It is worth noticing, however, that although both diseases are heterogenous, T2D is probably more heterogenous than T1D and can be classified into multiple subtypes according to clinical parameters and phenotype, and genetics most likely play an important underlying role in this (Udler, 2019). Future studies comparing T1D genetics with the various subclasses of T2D categorized by genetic profiles would be of interest.

A limitation of our study may be that the GWAS datasets used to define the disease-associated loci are from European populations only. We therefore might have missed potential GWAS signals that could be present in other ethnicities. Another limitation is that it was not possible to use PPIs obtained from human islets as such data currently does not exist. It is therefore not possible to apply tissue specificity at this point. In general, however, PPIs are not tissue-specific, though, but, obviously, rely on the expression of the protein-coding genes that interact at the protein level. We applied islet gene expression filtering that indirectly added some tissue specificity for the PPI analyses, but it would have been further advantageous if PPI data for human islets existed. Additional studies are highly warranted to validate the results and to explore the roles of the identified common candidate genes for normal and dysfunctional islet mass.

In summary, by a dual systems genetics approach, we report the identification of novel plausible causal T1D and T2D risk genes that are common between both diabetes forms. Our study further suggests that some genes located in T1D and T2D risk loci interact in shared islet networks where they regulate critical cellular functions such as cell cycle processes and lipid metabolism in human islets. From our findings novel testable hypotheses can be formulated thereby setting the groundwork for future experimental follow up and functional characterization of the shared and interacting T1D and T2D candidate genes in *in vitro* and *in vivo* models. Moreover, it would be imperative to experimentally validate the identified PPIs in human islets and in β-cells by appropriate methods. These studies are highly warranted as they could shed further light onto causal and pathogenic mechanisms and offer new clues about how genetic factors set the scene for immune- and metabolic stress-mediated β-cell loss in T1D and T2D.

## Data Availability Statement

The original contributions presented in the study are included in the article/Supplementary Material, further inquiries can be directed to the corresponding author.

## Author Contributions

JS suggested the overall hypothesis. SK and JS designed the study and researched the data. SK, JS, and AO drafted the manuscript. FP and AM revised the manuscript critically for important intellectual content. All authors contributed to the article and approved the submitted version.

## Conflict of Interest

The authors declare that the research was conducted in the absence of any commercial or financial relationships that could be construed as a potential conflict of interest.

## Footnotes

^1^*I*mmunobase. Available online at: https://www.immunobase.org/.

^2^IPA (QIAGEN Inc). Available online at: https://digitalinsights.qiagen.com/plugins/ingenuity-pathway-analysis/.

## Supplementary Material

Supplementary File 1.

Supplementary Table 1.1 Annotation of islet eQTL signals and biotype of islet eQTL genes. The islet eQTL associations were retrieved from Viñuela et al. (2020). Both exon and gene-level *cis*-eQTLs corresponding to 4,312 and 6,039 genes, respectively (FDR<1%; *cis* defined as within 1 Mb of the transcription start site [TSS]), were combined that resulted in a total of 10,108 islet eQTL associations for 6,618 islet eQTL genes.

Supplementary Table 1.2 T1D-associated SNPs as islet eQTLs. The table lists 55 T1D-associated SNPs that act as islet eQTLs. T1D GWAS summary statistics were retrieved from Onengut-Gumuscu et al. (2015). All nominally associated SNPs (*p* < 0.05) were compared against the significant exon and gene level islet eQTLs from Viñuela et al. (2020). OR, log odds ratio for the effect allele. T1D GWAS Alleles (Min>Maj); Islet eQTL Alleles (Ref>Alt).

Supplementary Table 1.3 T2D-associated SNPs as islet eQTLs. The table lists 19 T2D-associated SNPs that act as islet eQTLs. The T2D GWAS summary statistics were retrieved from Mahajan et al. (2019) (European BMI adjusted dataset). All nominally associated SNPs (*p* < 0.05) were compared against the significant exon and gene level islet eQTLs from Viñuela et al. (2020). Beta: log odds ratio for the effect allele; T2D GWAS Alleles (Effect allele > other allele); Islet eQTL Alleles (Ref>Alt).

Supplementary Table 1.4 Pathway-based annotation analysis of the “T1D-T2D islet eQTL interaction network.” The pathway-based annotations were performed using KEGG, Reactome, and Wiki pathway annotations in ClueGo app in Cytoscape. P-values were corrected with Bonferroni step down.

Supplementary Table 1.5 Shared genes in the “cytokine and palmitate islet interaction network”.

Supplementary Table 1.6 Pathway-based annotation analysis of the “cytokine and palmitate islet interaction network.” The pathway-based annotation was performed using KEGG, Reactome, and Wiki pathway annotations in ClueGo app in Cytoscape. P-values are corrected with Bonferroni step down.

Supplementary Figure 1 Pathway based functional annotation of “T1D-T2D islet eQTL interaction network” (117 nodes with node degree ≥1) using CytoScape plugin ClueGO.

Supplementary Figure 2 Pathway based functional annotation of “cytokine and palmitate islet interaction network” (181 nodes with node degree ≥1) using CytoScape plugin ClueGO.

Supplementary File 2.

Supplementary Table 2.1 Annotation of islet eQTL SNPs based on islet regulome features. A total of 1,282 islet eQTL SNPs intersect with islet regulome features derived from Miguel-Escalada et al. (2019). All the coordinates are based on the GRCh37 version of the human genome.

Supplementary Table 2.2 Annotation of T1D-associated SNPs in LD with islet eQTL SNPs based on islet regulome features. 140 T1D-associated SNPs in LD with islet eQTLs intersect with islet regulome features derived from Miguel-Escalada et al. (2019). All the coordinates are based on the GRCh37 version of the human genome.

Supplementary Table 2.3 Annotation of T2D-associated SNPs in LD with islet eQTL SNPs based on islet regulome features. 22 T2D SNPs in LD with islet eQTLs intersect with islet regulome features derived from Miguel-Escalada et al. (2019). All the coordinates are based on the GRCh37 version of the human genome.

Supplementary File 3. High resolution view of the 'T1D-T2D islet eQTL interaction network' as visualized by Cytoscape. T1D (*n* = 204) and T2D genes (*n* = 192) are shown as cyan and red nodes, respectively. The edges (blue lines) represent the physicial interactions between the nodes. The network consists of 361 nodes, of which 9 are shared (shown in green).

Supplementary File 4. High resolution view of the ‘cytokine and palmitate islet interaction network’ as visualized by Cytoscape. The T1D loci genes subject to cytokine regulation (*n* = 191) and T2D loci genes subject to palmitate regulation (*n* = 187) are shown in cyan and red nodes, respectively. The edges (blue lines) represent the physicial interactions between the nodes. The network consists of 372 nodes, of which 4 are shared (shown in green).
