# Supplementary material for: A Dual Systems Genetics Approach Identifies Common Genes, Networks, and Pathways for Type 1 and 2 Diabetes in Human Islets
Source: Front Genet. 2021 Mar 10;12:630109. doi: 10.3389/fgene.2021.630109 (PMC7987941; doi:10.3389/fgene.2021.630109)

**Supplementary Table 1.1** Annotation of islet eQTL signals and islet eQTL genes.

Islet eQTL associations were retrieved from Viñuela et al. (*Nat Commun* 11, 4912 (2020) doi: [10.1038/s41467-020-18581-8](https://doi.org/10.1038/s41467-020-18581-8)). Both exon and gene-level *cis*-eQTLs corresponding to 4,312 and 6,039 genes, respectively (FDR < 1%; *cis* defined as within 1Mb of the transcription start site), were combined to result in a total of 10,108 islet eQTL associations for 6,618 islet eQTL genes.

| **Gene type** | **Islet eQTL genes (count)** | **Islet eQTL signals (count)** |
| --- | --- | --- |
| LincRNA | 548 | 842 |
| Protein coding | 6,070 | 9,627 |
| **Total** | 6,618 | 10,469 (10,108 non-redundant) |

**Supplementary Table 1.2** T1D SNPs as islet eQTLs.

The table lists the 55 T1D-associated SNPs that act as islet eQTLs. T1D GWAS summary statistics were retrieved from Onengut-Gumuscu et al. (*Nat. Genet.* 47, 381–386 (2015) doi:[10.1038/ng.3245](https://doi.org/10.1038/ng.3245)). All nominally associated SNPs (p-value < 0.05) were compared against the significant exon and gene level islet eQTLs from Viñuela et al. (*Nat Commun* 11, 4912 (2020) doi: [10.1038/s41467-020-18581-8](https://doi.org/10.1038/s41467-020-18581-8)). OR: log odds ratio for the effect allele; T1D GWAS alleles (minor > major); Islet eQTL alleles (reference > alternative).

| **T1D GWAS** | | | | | | **Islet eQTLs** | | | |
| --- | --- | --- | --- | --- | --- | --- | --- | --- | --- |
| **SNP** | **Chr** | **SNP**  **start** | **T1D**  **p-value** | **OR** | **T1D GWAS alleles** | **Islet eQTL alleles** | **Gene name** | **Strand** | **Gene biotype** |
| rs7482891 | 11 | 2197112 | 1.28E-57 | 0.7261 | A>G | A>G | TH | - | protein_coding |
| rs679574 | 19 | 49206108 | 5.22E-14 | 0.8668 | C>G | C>G | FUT2 | + | protein_coding |
| rs3087876 | 16 | 11036624 | 3.75E-09 | 0.894 | A>G | A>G | CIITA | + | protein_coding |
| rs3087876 | 16 | 11036624 | 3.75E-09 | 0.894 | A>G | A>G | DEXI | - | protein_coding |
| rs6688827 | 1 | 113945989 | 8.32E-09 | 1.1286 | A>C | C>A | MAGI3 | + | protein_coding |
| rs870829 | 17 | 38068382 | 2.42E-08 | 1.1118 | A>C | A>C | GSDMB | - | protein_coding |
| rs160544 | 19 | 47234885 | 5.73E-07 | 0.865 | A>G | G>A | FKRP | + | protein_coding |
| rs72669177 | 4 | 123480484 | 9.15E-07 | 1.114 | A>C | C>A | KIAA1109 | + | protein_coding |
| rs3745609 | 19 | 47282240 | 5.36E-06 | 0.913 | G>A | A>G | FKRP | + | protein_coding |
| rs6565259 | 16 | 28898793 | 1.25E-05 | 0.9204 | C>T | T>C | EIF3CL | - | protein_coding |
| rs968567 | 11 | 61595564 | 3.79E-05 | 0.9021 | A>G | C>T | FADS2 | + | protein_coding |
| rs12719056 | 7 | 50510076 | 5.06E-05 | 0.9259 | T>C | T>C | FIGNL1 | - | protein_coding |
| rs418821 | 19 | 49199804 | 7.23E-05 | 0.8807 | G>C | G>C | FUT2 | + | protein_coding |
| rs7803247 | 7 | 50540743 | 9.83E-05 | 0.9287 | T>C | T>C | FIGNL1 | - | protein_coding |
| rs2952152 | 17 | 37832735 | 0.000145 | 0.9268 | T>C | T>C | PGAP3 | - | protein_coding |
| rs2941506 | 17 | 37833035 | 0.000177 | 0.9277 | A>G | A>G | PGAP3 | - | protein_coding |
| rs1862705 | 16 | 75346788 | 0.000194 | 1.0855 | C>G | C>G | CFDP1 | - | protein_coding |
| rs1862705 | 16 | 75346788 | 0.000194 | 1.0855 | C>G | C>G | RP11-77K12.1 | - | protein_coding |
| rs2236441 | 21 | 45755360 | 0.000219 | 1.0887 | T>C | C>T | C21orf2 | - | protein_coding |
| rs11640470 | 16 | 75299437 | 0.000282 | 1.0714 | A>G | A>G | BCAR1 | - | protein_coding |
| rs11168249 | 12 | 48208368 | 0.000345 | 1.0704 | C>T | T>C | HDAC7 | - | protein_coding |
| rs28400014 | 19 | 49250657 | 0.000421 | 0.9352 | G>C | C>G | RASIP1 | - | protein_coding |
| rs6599390 | 4 | 956047 | 0.000465 | 0.9324 | A>G | A>G | SLC26A1 | - | protein_coding |
| rs73392899 | 22 | 30201381 | 0.000578 | 0.8624 | C>T | C>T | ASCC2 | - | protein_coding |
| rs775451 | 12 | 70112707 | 0.001542 | 0.9094 | C>T | A>G | AC025263.3 | + | protein_coding |
| rs4840581 | 8 | 11635569 | 0.001901 | 0.9427 | T>C | T>C | NEIL2 | + | protein_coding |
| rs41284471 | 10 | 6626214 | 0.002353 | 0.9296 | A>G | G>A | PRKCQ-AS1 | + | lincRNA |
| rs11688877 | 2 | 100724445 | 0.003093 | 0.9315 | C>T | T>C | AFF3 | - | protein_coding |
| rs2069502 | 12 | 58144665 | 0.003178 | 0.9427 | T>C | C>T | TSFM | + | protein_coding |
| rs3825078 | 12 | 58089413 | 0.003732 | 0.9436 | A>T | A>T | OS9 | + | protein_coding |
| rs10877012 | 12 | 58162085 | 0.004372 | 0.9446 | T>G | G>T | RP11-571M6.15 | + | protein_coding |
| rs1736560 | 1 | 171059150 | 0.00596 | 1.0565 | G>C | C>G | FMO3 | + | protein_coding |
| rs10877015 | 12 | 58167788 | 0.00596 | 0.9465 | G>A | A>G | METTL21B | + | protein_coding |
| rs17508449 | 1 | 114085145 | 0.010128 | 0.9305 | T>C | C>T | PHTF1 | - | protein_coding |
| rs7578575 | 2 | 25488819 | 0.010772 | 1.0523 | A>T | T>A | DNMT3A | - | protein_coding |
| rs61741425 | 7 | 105148593 | 0.011678 | 0.8403 | A>G | G>A | PUS7 | - | protein_coding |
| rs3736328 | 19 | 18263703 | 0.012419 | 1.046 | C>G | C>G | MAST3 | + | protein_coding |
| rs7063 | 5 | 96110211 | 0.014286 | 0.9522 | T>A | A>T | ERAP1 | - | protein_coding |
| rs9862534 | 3 | 49598064 | 0.015158 | 1.0523 | T>C | T>C | DAG1 | + | protein_coding |
| rs17294280 | 15 | 67468285 | 0.015158 | 1.0523 | G>A | A>G | SMAD3 | + | protein_coding |
| rs4521268 | 3 | 49137904 | 0.015476 | 1.0471 | G>T | G>T | WDR6 | + | protein_coding |
| rs1057569 | 5 | 96109610 | 0.016395 | 0.9531 | A>G | G>A | CAST | + | protein_coding |
| rs10877013 | 12 | 58165085 | 0.016395 | 0.9531 | T>C | C>T | TSFM | + | protein_coding |
| rs5029444 | 7 | 23737795 | 0.017864 | 0.956 | T>C | T>C | FAM221A | + | protein_coding |
| rs3745733 | 19 | 49132634 | 0.019631 | 0.9389 | A>G | G>A | CA11 | - | protein_coding |
| rs2059818 | 19 | 52882520 | 0.02057 | 0.957 | C>T | T>C | ZNF880 | + | protein_coding |
| rs2070896 | 16 | 31384554 | 0.023626 | 0.9579 | C>T | T>C | ZNF48 | + | protein_coding |
| rs9834003 | 3 | 49216472 | 0.025215 | 1.0481 | T>C | T>C | RHOA | - | protein_coding |
| rs4021 | 19 | 49253261 | 0.025215 | 0.9541 | G>A | A>G | FUT1 | - | protein_coding |
| rs55839314 | 1 | 161329682 | 0.025297 | 1.0887 | A>G | G>A | RP11-122G18.5 | + | lincRNA |
| rs4148974 | 21 | 44323720 | 0.026081 | 0.9148 | T>C | C>T | NDUFV3 | + | protein_coding |
| rs8048078 | 16 | 70737106 | 0.027069 | 1.0429 | T>C | T>C | MTSS1L | - | protein_coding |
| rs919270 | 19 | 52668553 | 0.029712 | 0.9512 | T>C | A>G | ZNF836 | - | protein_coding |
| rs11172344 | 12 | 58193448 | 0.03026 | 0.9618 | C>T | T>C | METTL21B | + | protein_coding |
| rs467812 | 22 | 29940949 | 0.034763 | 0.9627 | A>G | G>A | NIPSNAP1 | - | protein_coding |
| rs198464 | 11 | 61521621 | 0.039825 | 0.9637 | T>C | G>A | RP11-467L20.10 | - | lincRNA |
| rs268858 | 2 | 65471137 | 0.040108 | 0.9618 | T>A | T>A | ACTR2 | + | protein_coding |

**Supplementary Table 1.3** T2D SNPs as islet eQTLs.

The table lists the 19 T2D SNPs that act as islet eQTLs. The T2D GWAS summary statistics were retrieved from Mahajan et al. (*Nat Genet* 50, 1505–1513. doi:[10.1038/s41588-018-0241-6](https://doi.org/10.1038/s41588-018-0241-6)) (European BMI-adjusted dataset). All nominally associated SNPs (p-value < 0.05) were compared against the significant exon and gene level islet eQTLs from Viñuela et al. (*Nat Commun* 11, 4912 (2020) doi: [10.1038/s41467-020-18581-8](https://doi.org/10.1038/s41467-020-18581-8)). Beta: log odds ratio for the effect allele; T2D GWAS alleles (effect allele > other allele); Islet eQTL alleles (reference > alternative).

| **T2D GWAS** | | | | | | **islet eQTLs** | | | |
| --- | --- | --- | --- | --- | --- | --- | --- | --- | --- |
| **SNP** | **Chr** | **SNP**  **START** | **T2D**  **p-value** | **Beta** | **T2D GWAS alleles** | **Islet eQTL**  **alleles** | **Gene name** | **Strand** | **Gene biotype** |
| rs10995311 | 10 | 64564934 | 8.50E-05 | 0.0208 | C>G | C>G | ADO | + | protein_coding |
| rs8012 | 19 | 13010520 | 0.0001311 | 0.0397 | A>G | A>G | GCDH | + | protein_coding |
| rs1209879 | 8 | 146076708 | 0.001315 | -0.0242 | T>C | C>T | ZNF34 | - | protein_coding |
| rs17856580 | 16 | 27246617 | 0.002621 | -0.0266 | T>C | T>C | KDM8 | + | protein_coding |
| rs3733227 | 4 | 83582211 | 0.003082 | 0.0193 | C>G | C>G | SCD5 | - | protein_coding |
| rs3130981 | 6 | 31083813 | 0.003272 | -0.0214 | T>C | T>C | PSORS1C1 | + | protein_coding |
| rs1805081 | 18 | 21140432 | 0.009315 | 0.0182 | T>C | T>C | NPC1 | - | protein_coding |
| rs61574510 | 19 | 52520607 | 0.009829 | 0.0227 | T>C | C>T | ZNF614 | - | protein_coding |
| rs11761888 | 7 | 130007381 | 0.01205 | 0.0191 | T>C | T>C | CPA5 | + | protein_coding |
| rs10134537 | 14 | 24760764 | 0.01469 | -0.036 | A>G | G>A | DHRS1 | - | protein_coding |
| rs7216284 | 17 | 4463699 | 0.01638 | -0.0189 | A>G | G>A | GGT6 | - | protein_coding |
| rs143356584 | 10 | 25241502 | 0.0165 | -0.0716 | T>C | C>T | PRTFDC1 | - | protein_coding |
| rs117582579 | 17 | 28512405 | 0.02162 | -0.0759 | A>G | G>A | NSRP1 | + | protein_coding |
| rs8050871 | 16 | 71509796 | 0.0308 | -0.0115 | C>G | C>G | ZNF19 | - | protein_coding |
| rs6897513 | 5 | 35641582 | 0.03552 | -0.0175 | A>C | A>C | SPEF2 | + | protein_coding |
| rs2277339 | 12 | 57146069 | 0.03567 | -0.0165 | T>G | T>G | PRIM1 | - | protein_coding |
| rs10282929 | 8 | 144681777 | 0.03713 | -0.0236 | T>G | G>T | NAPRT1 | - | protein_coding |
| rs8141797 | 22 | 24582041 | 0.03734 | 0.027 | A>G | A>G | SUSD2 | + | protein_coding |
| rs3195676 | 5 | 34008100 | 0.04576 | 0.0179 | T>C | C>T | AMACR | - | protein_coding |

**Supplementary Table 1.4** Pathway-based annotation analysis of the ‘T1D-T2D islet eQTL interaction network’.

Pathway-based annotation was performed using KEGG, Reactome and WikiPathway annotations in ClueGo app in Cytoscape. P-values were corrected using Bonferroni step down.

| **ID** | **Term** | **Term**  **p-value** | **Group**  **p-value** | **Groups** | **% Associated genes** | **No. of genes** | **Associated genes found** |
| --- | --- | --- | --- | --- | --- | --- | --- |
| KEGG:00600 | Sphingolipid metabolism | 2.94E-02 | 1.26E-02 | Group0 | 6.38 | 3 | CERS2, GBA, GLB1 |
| R-HSA:381340 | Transcriptional regulation of white adipocyte differentiation | 2.47E-02 | 1.10E-02 | Group1 | 4.76 | 4 | CDK8, MED1, MED28, MED31 |
| WP:45 | G1 to S cell cycle control | 4.97E-02 | 1.99E-02 | Group2 | 4.69 | 3 | CDC25A, POLA2, PRIM1 |
| R-HSA:2151201 | Transcriptional activation of mitochondrial biogenesis | 4.13E-02 | 1.10E-02 | Group3 | 5.36 | 3 | ACSS2, ATF2, MED1 |
| WP:3996 | Ethanol effects on histone modifications | 1.26E-02 | 1.10E-02 | Group3 | 9.68 | 3 | ACSS2, ATF2, HDAC7 |
| KEGG:04520 | Adherens junction | 3.95E-02 | 1.20E-02 | Group4 | 4.23 | 3 | RHOA, SMAD3, TCF7L2 |
| R-HSA:170834 | Signaling by TGF-beta Receptor Complex | 2.84E-02 | 1.20E-02 | Group4 | 4.11 | 3 | CDK8, RHOA, SMAD3 |
| WP:2324 | AGE/RAGE pathway | 4.32E-02 | 1.20E-02 | Group4 | 4.55 | 3 | ATF2, RHOA, SMAD3 |
| WP:4216 | Chromosomal and microsatellite instability in colorectal cancer | 1.47E-02 | 1.20E-02 | Group4 | 4.05 | 3 | RHOA, SMAD3, TCF7L2 |
| WP:4535 | Envelope proteins and their potential roles in EDMD physiopathology | 3.17E-02 | 1.20E-02 | Group4 | 6.52 | 3 | PLEC, RHOA, SMAD3 |

**Supplementary Table 1.5** Shared genes among the cytokine- and palmitate-regulated T1D/T2D loci genes.

| **Gene ID** | **Gene name** | **Islet eQTL SNP** |
| --- | --- | --- |
| ENSG00000100325 | ASCC2 | rs12628234,rs73392899 |
| ENSG00000106049 | HIBADH | rs62454876,rs11767324 |
| ENSG00000172575 | RASGRP1 | NA |
| ENSG00000266028 | SRGAP2 | NA |

**Supplementary Table 1.6** Pathway-based annotation analysis of the ‘cytokine and palmitate islet interaction network’.

Pathway-based annotation was performed using KEGG, Reactome, and WikiPathway annotations in ClueGo app in Cytoscape. P-values were corrected using Bonferroni step down.

| **ID** | **Term** | **Term**  **p-value** | **Group**  **p-value** | **Groups** | **% Associated genes** | **No. of genes** | **Associated genes found** |
| --- | --- | --- | --- | --- | --- | --- | --- |
| R-HSA:9013694 | Signaling by NOTCH4 | 3.87E-02 | 7.90E-04 | Group0 | 7.32 | 6 | ACTA2, FBXW7, NOTCH2, PSMB1, PSMB8, PSMB9 |
| WP:2864 | Apoptosis-related network due to altered Notch3 in ovarian cancer | 4.00E-02 | 4.12E-04 | Group1 | 9.26 | 5 | APOE, AXIN1, ERBB3, ERN1, IL7R |
| KEGG:04145 | Phagosome | 8.18E-03 | 1.85E-08 | Group2 | 5.92 | 9 | HLA-DMA, HLA-DMB, HLA-DPA1, HLA-DRA, HLA-DRB5, ITGB3, TAP1, TAP2, TUBA4A |
| KEGG:04612 | Antigen processing and presentation | 4.10E-04 | 1.85E-08 | Group2 | 10.26 | 8 | CIITA, HLA-DMA, HLA-DMB, HLA-DPA1, HLA-DRA, HLA-DRB5, TAP1, TAP2 |
| KEGG:04640 | Hematopoietic cell lineage | 2.37E-03 | 1.85E-08 | Group2 | 8.08 | 8 | HLA-DMA, HLA-DMB, HLA-DPA1, HLA-DRA, HLA-DRB5, IL2RA, IL7R, ITGB3 |
| KEGG:04658 | Th1 and Th2 cell differentiation | 1.08E-02 | 1.85E-08 | Group2 | 7.61 | 7 | HLA-DMA, HLA-DMB, HLA-DPA1, HLA-DRA, HLA-DRB5, IL2RA, NOTCH2 |
| KEGG:04659 | Th17 cell differentiation | 2.70E-02 | 1.85E-08 | Group2 | 6.54 | 7 | HLA-DMA, HLA-DMB, HLA-DPA1, HLA-DRA, HLA-DRB5, IL2RA, RARA |
| KEGG:04672 | Intestinal immune network for IgA production | 2.68E-02 | 1.85E-08 | Group2 | 10.20 | 5 | HLA-DMA, HLA-DMB, HLA-DPA1, HLA-DRA, HLA-DRB5 |
| KEGG:04940 | Type I diabetes mellitus | 1.16E-03 | 1.85E-08 | Group2 | 13.95 | 6 | HLA-DMA, HLA-DMB, HLA-DPA1, HLA-DRA, HLA-DRB5, PTPRN2 |
| KEGG:05140 | Leishmaniasis | 2.83E-02 | 1.85E-08 | Group2 | 7.79 | 6 | EEF1A2, HLA-DMA, HLA-DMB, HLA-DPA1, HLA-DRA, HLA-DRB5 |
| KEGG:05145 | Toxoplasmosis | 3.43E-02 | 1.85E-08 | Group2 | 6.25 | 7 | CIITA, HLA-DMA, HLA-DMB, HLA-DPA1, HLA-DRA, HLA-DRB5, SOCS1 |
| KEGG:05150 | Staphylococcus aureus infection | 1.38E-02 | 1.85E-08 | Group2 | 7.29 | 7 | HLA-DMA, HLA-DMB, HLA-DPA1, HLA-DRA, HLA-DRB5, ICAM1, KRT40 |
| KEGG:05152 | Tuberculosis | 2.73E-02 | 1.85E-08 | Group2 | 5.00 | 9 | CEBPG, CIITA, CTSD, HLA-DMA, HLA-DMB, HLA-DPA1, HLA-DRA, HLA-DRB5, LSP1 |
| KEGG:05164 | Influenza A | 3.52E-03 | 1.85E-08 | Group2 | 5.88 | 10 | CIITA, HLA-DMA, HLA-DMB, HLA-DPA1, HLA-DRA, HLA-DRB5, ICAM1, IKBKE, KPNA2, OAS3 |
| KEGG:05166 | Human T-cell leukemia virus 1 infection | 6.00E-03 | 1.85E-08 | Group2 | 5.02 | 11 | ADCY5, CDKN2C, FOSL1, HLA-DMA, HLA-DMB, HLA-DPA1, HLA-DRA, HLA-DRB5, ICAM1, IL2RA, MYC |
| KEGG:05169 | Epstein-Barr virus infection | 8.03E-05 | 1.85E-08 | Group2 | 6.47 | 13 | DDB2, HLA-DMA, HLA-DMB, HLA-DPA1, HLA-DRA, HLA-DRB5, ICAM1, IKBKE, MYC, OAS3, TAP1, TAP2, TNFAIP3 |
| KEGG:05310 | Asthma | 3.13E-03 | 1.85E-08 | Group2 | 16.13 | 5 | HLA-DMA, HLA-DMB, HLA-DPA1, HLA-DRA, HLA-DRB5 |
| KEGG:05320 | Autoimmune thyroid disease | 3.74E-02 | 1.85E-08 | Group2 | 9.43 | 5 | HLA-DMA, HLA-DMB, HLA-DPA1, HLA-DRA, HLA-DRB5 |
| KEGG:05330 | Allograft rejection | 8.29E-03 | 1.85E-08 | Group2 | 13.16 | 5 | HLA-DMA, HLA-DMB, HLA-DPA1, HLA-DRA, HLA-DRB5 |
| KEGG:05332 | Graft-versus-host disease | 1.17E-02 | 1.85E-08 | Group2 | 12.20 | 5 | HLA-DMA, HLA-DMB, HLA-DPA1, HLA-DRA, HLA-DRB5 |
| KEGG:05416 | Viral myocarditis | 7.11E-04 | 1.85E-08 | Group2 | 11.67 | 7 | HLA-DMA, HLA-DMB, HLA-DPA1, HLA-DRA, HLA-DRB5, ICAM1, RAC2 |
| R-HSA:2132295 | MHC class II antigen presentation | 1.06E-02 | 1.85E-08 | Group2 | 6.50 | 8 | AP1B1, AP2M1, CTSD, HLA-DMB, HLA-DPA1, HLA-DRA, HLA-DRB5, TUBA4A |
| R-HSA:877300 | Interferon gamma signaling | 1.08E-02 | 1.85E-08 | Group2 | 7.61 | 7 | CIITA, HLA-DPA1, HLA-DRA, HLA-DRB5, ICAM1, OAS3, SOCS1 |
| WP:619 | Type II interferon signaling (IFNG) | 7.40E-03 | 1.85E-08 | Group2 | 13.51 | 5 | CIITA, ICAM1, PSMB9, SOCS1, TAP1 |

**Supplementary Figure S1** Pathway-based functional annotation of the ‘T1D-T2D islet eQTL interaction network’ (117 nodes with node degree ≥ 1) using CytoScape plugin ClueGO.


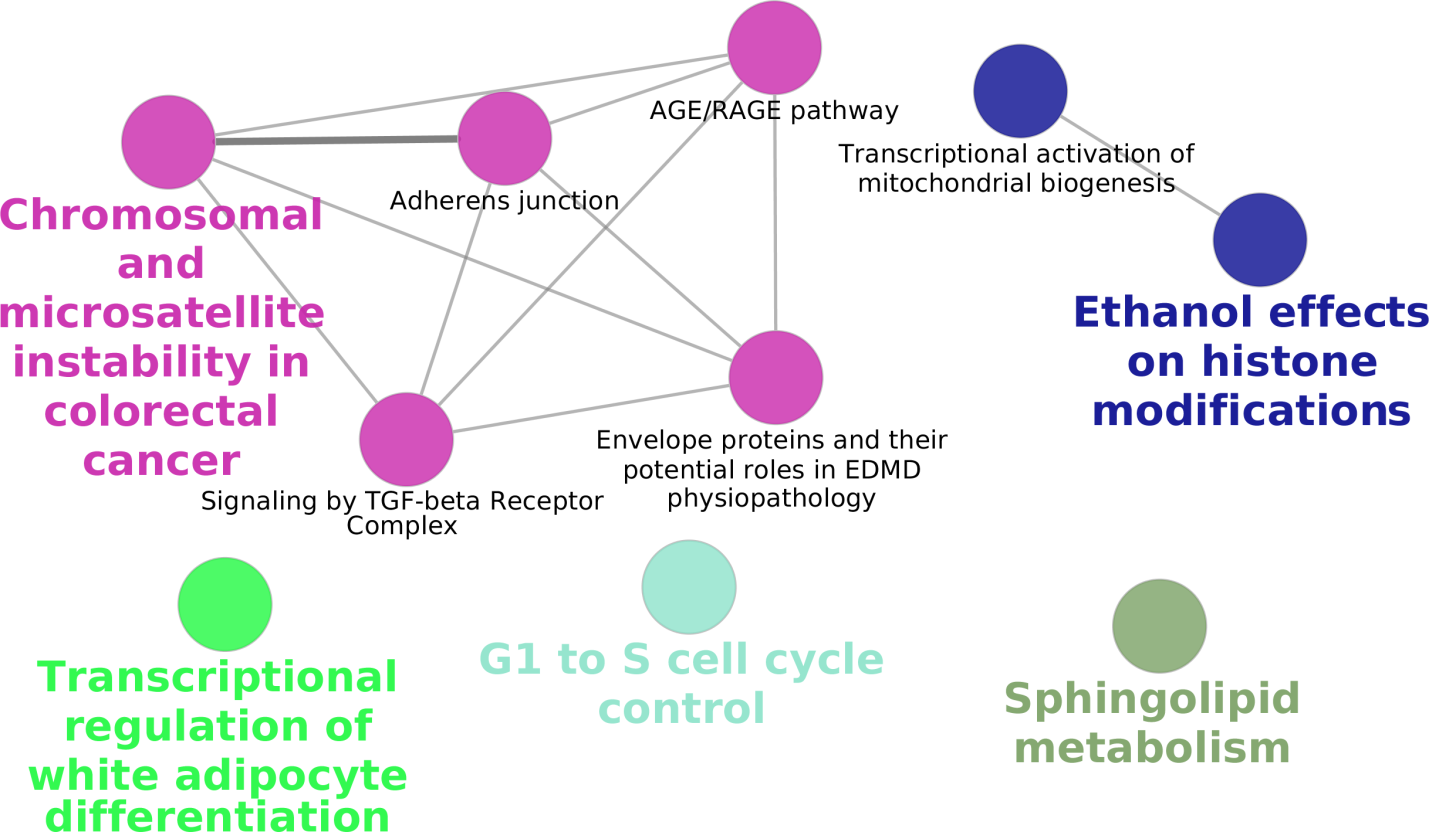


**Supplementary Figure S2** Pathway-based functional annotation of the ‘cytokine and palmitate islet interaction network’ (181 nodes with node degree ≥ 1) using CytoScape plugin ClueGO.


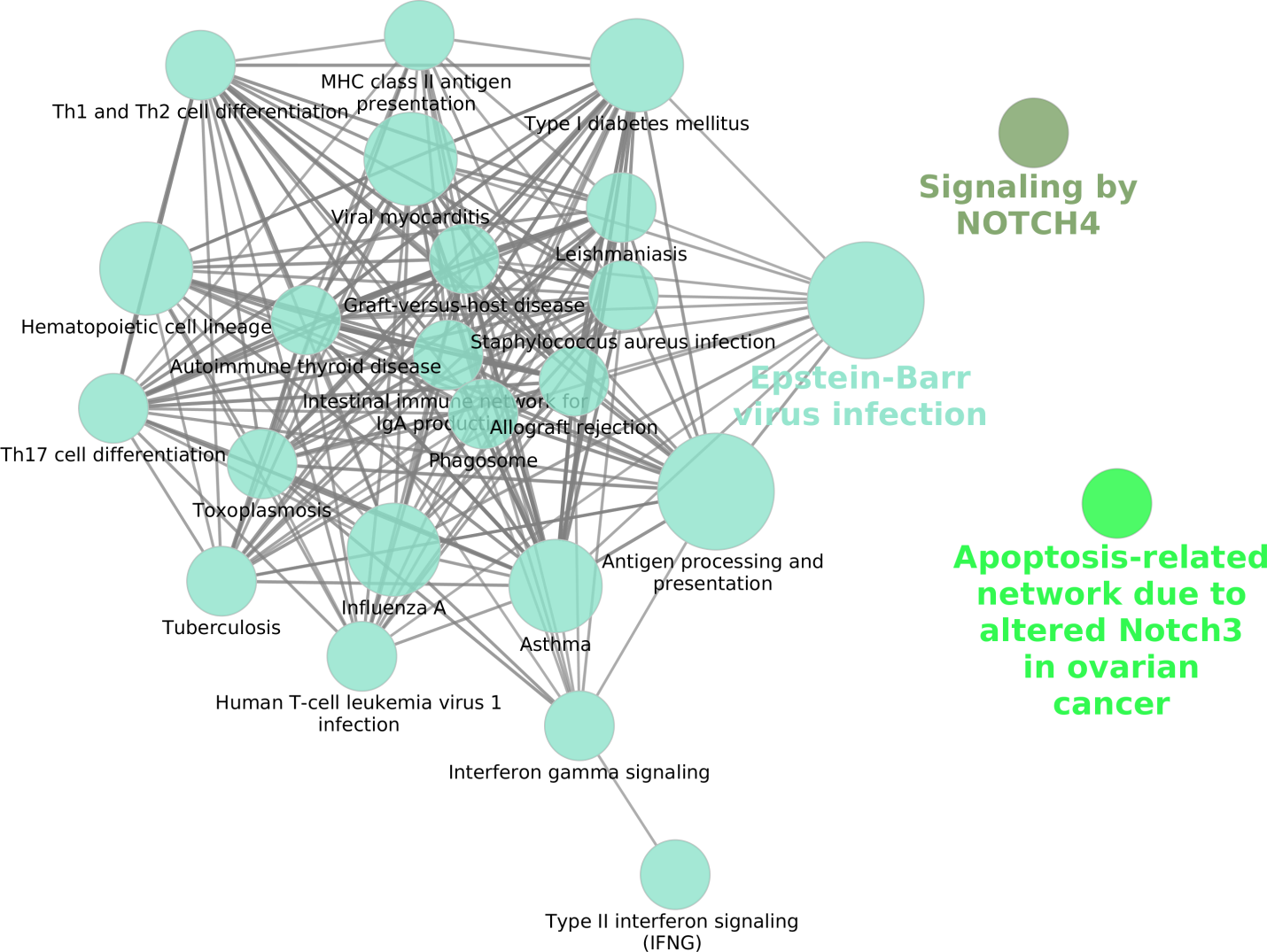

Supplement: Supplementary File 1. [file Presentation_1.zip › Supplementary File 1.docx]
