# Supplementary figures and images for: A Dual Systems Genetics Approach Identifies Common Genes, Networks, and Pathways for Type 1 and 2 Diabetes in Human Islets
Source: Front Genet. 2021 Mar 10;12:630109. doi: 10.3389/fgene.2021.630109 (PMC7987941; doi:10.3389/fgene.2021.630109)

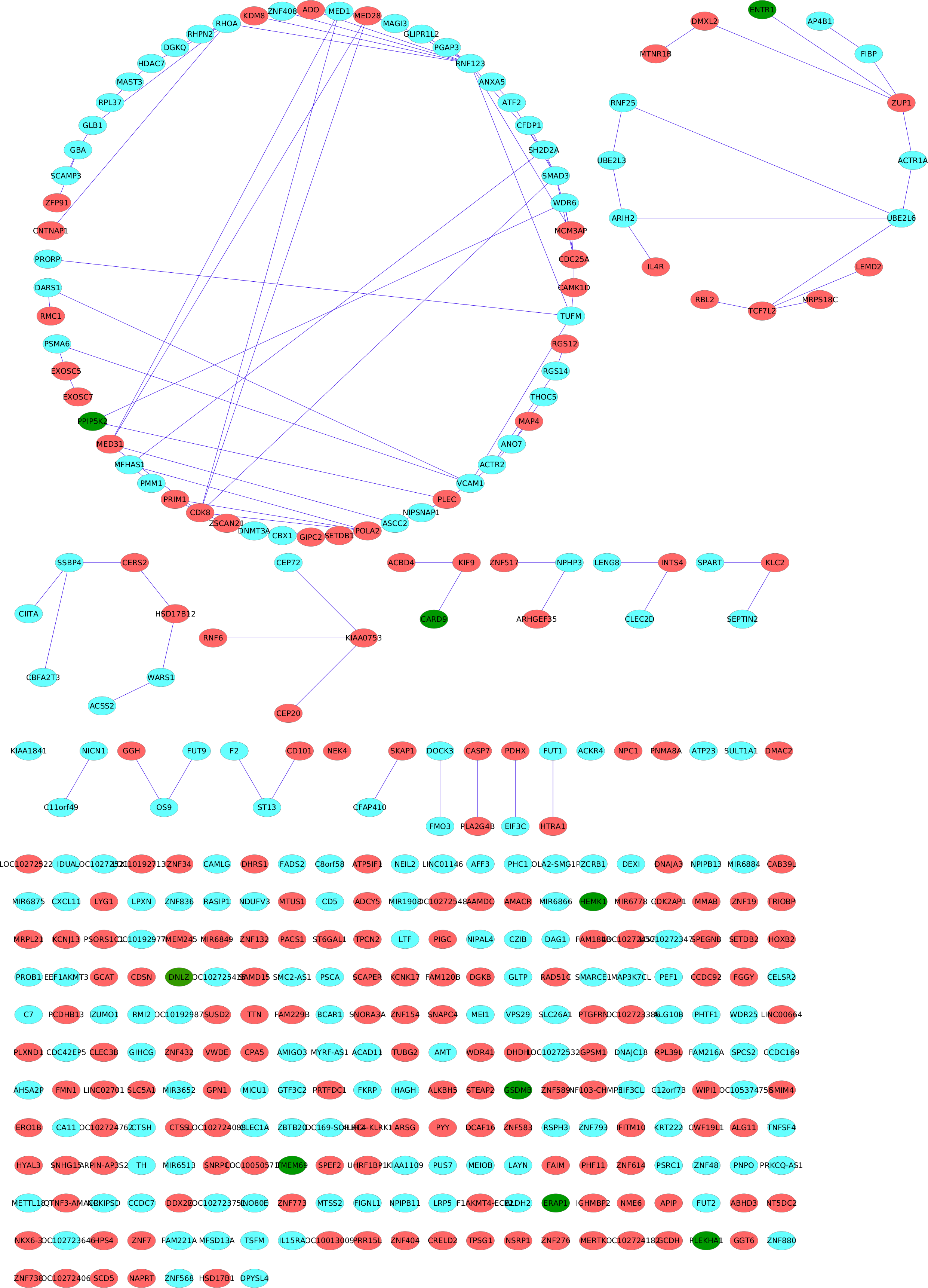

Supplement: Supplementary File 1. [file Presentation_1.zip › Supplementary File 3.TIFF]

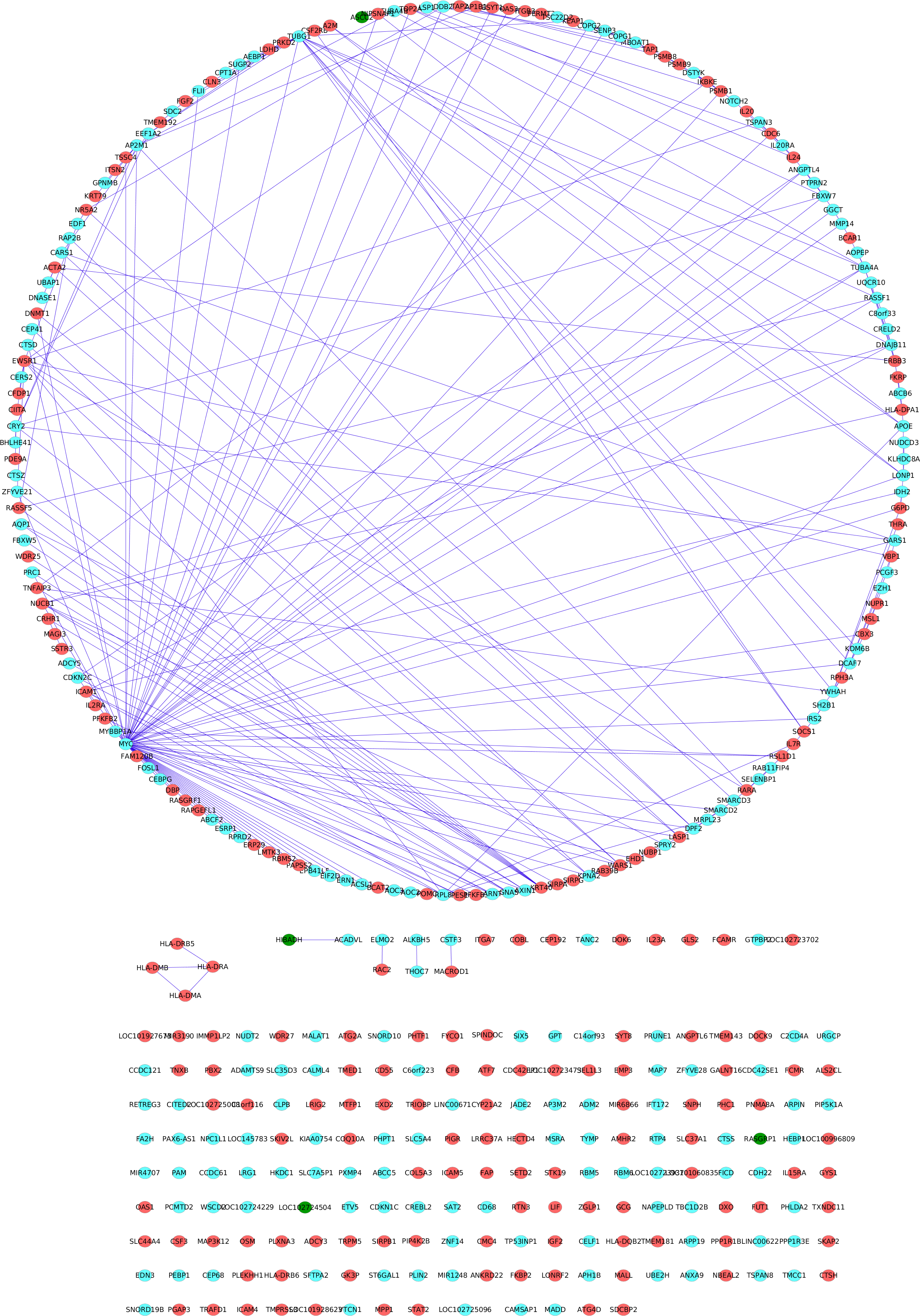

Supplement: Supplementary File 1. [file Presentation_1.zip › Supplementary File 4.TIFF]
